# Critical care nephrology: opportunities for implementing green practices

**DOI:** 10.3389/fmed.2025.1635718

**Published:** 2025-10-01

**Authors:** Alejandra Molano-Triviño, Lilia Maria Rizo-Topete, Eduardo Zúñiga, Juan Camilo Castellanos-De la Hoz, Akash Nayak Karopadi

**Affiliations:** ^1^Department of Nephrology, Fundacion Cardioinfantil La Cardio, Bogotá, Colombia; ^2^Department of Nephrology, Universidad Autonoma de Nuevo Leon, San Nicolás de los Garza, Mexico; ^3^Alira Health, Boston, MA, United States

**Keywords:** green nephrology, dialysis, water waste, plastic, critical care

## Abstract

The intersection between climate change, healthcare, and nephrology is becoming increasingly evident. Globally, healthcare systems contribute approximately 4.4% of greenhouse gas emissions, with intensive care units (ICUs) representing some of the most resource-intensive hospital areas. Within this environment, critical care nephrology plays a central role, particularly in managing acute kidney injury (AKI) and delivering renal replacement therapies (RRT) such as hemodialysis, continuous renal replacement therapy (CRRT), and peritoneal dialysis. Nephrology interventions in the ICU, while life-saving, are associated with high environmental costs, including significant water consumption, energy use, and the production of large volumes of medical waste For instance, a single hemodialysis session can consume over 500 liters of water, while CRRT in critically ill patients may require 10 to 15 large plastic bags daily, most of which are incinerated as hazardous waste Nephrology has pioneered “Green Nephrology” initiatives focused on reducing the ecological footprint of chronic dialysis through water reuse, optimized dialysis fluid flows, and material recycling (8, 11), however, similar structured strategies for sustainability within critical care nephrology remain underdeveloped. This article explores the environmental challenges of nephrology practice in ICUs, highlights existing gaps, and proposes opportunities, including artificial intelligence (AI) to promote sustainable, high-quality kidney care for critically ill patients.

## Introduction

The intersection between climate change, healthcare, and nephrology is becoming increasingly evident. Globally, healthcare systems contribute approximately 4.4% of greenhouse gas emissions, with intensive care units (ICUs) representing some of the most resource-intensive hospital areas (1). Within this environment, critical care nephrology plays a central role, particularly in managing acute kidney injury (AKI) and delivering renal replacement therapies (RRT) such as hemodialysis, continuous renal replacement therapy (CRRT), and peritoneal dialysis (2, 3).

Nephrology interventions in the ICU, while life-saving, are associated with high environmental costs, including significant water consumption, energy use, and the production of large volumes of medical waste (4, 5).

For instance, a single hemodialysis session can consume over 500 liters of water, while CRRT in critically ill patients may require 10 to 15 large plastic bags daily, most of which are incinerated as hazardous waste (3, 6–8).

The COVID-19 pandemic further amplified medical waste generation, especially from the extensive use of single-use plastics and personal protective equipment (PPE), highlighting the urgency of integrating sustainability into critical care nephrology (9, 10).

Nephrology has pioneered “Green Nephrology” initiatives focused on reducing the ecological footprint of chronic dialysis through water reuse, optimized dialysis fluid flows, and material recycling (8, 11), however, similar structured strategies for sustainability within critical care nephrology remain underdeveloped.

This article explores the environmental challenges of nephrology practice in ICUs, highlights existing gaps, and proposes opportunities, including artificial intelligence (AI) to promote sustainable, high-quality kidney care for critically ill patients.

### Medical waste and environmental burden in critical care nephrology

ICUs generate substantial volumes of heterogeneous medical waste due to the complexity of care, infection control measures, and the reliance on disposable equipment. Studies estimate that approximately 85% of general hospital waste is non-hazardous and potentially recyclable, yet most is discarded as general waste due to inadequate segregation—this figure applies to healthcare settings broadly but underscores missed opportunities within ICUs (12).

In critical care nephrology, waste originates primarily from RRT, vascular access procedures, fluid management, and associated consumables like personal protection disposables.

A vast amount of single-use plastics are discarded in all dialysis modalities in ICU as well as in chronic scenarios: In CRRT from circuits, replacement fluids and effluent bags, when using intermittent hemodialysis, there are also single-use plastics from circuits, filters and acid/bicarbonate gallons and remarkably Water consumption exceeding 100 liters per hemodialysis session (11). Discarded plastics imply incineration of contaminated materials, contributing to air pollution and greenhouse gas emissions (13, 14).

A “Green critical care nephrology” model requires an expert and mature team between hospital manager, ICU and nephrology to adapt constantly the well-known strategies for sustainability, not just the well-known Reduce, Reuse, recycle but also rethinking and repairing.

Healthcare’s ethical responsibility extends beyond individual patient care to include planetary health. Advancing sustainable critical care nephrology aligns clinical excellence with environmental stewardship, representing an essential evolution for modern medicine.

Potential scenarios of sustainability should be evaluated in case scenarios, but in general for critical care nephrology include:

#### Reduce

There are many traditional efforts to reduce waste of resources in hospitals in general and in ICU:

- Energy Consumption Reduction: the use of high-efficiency lighting, turning off electronic equipment when not in use, and promoting renewable energy use.
- Single use waste: Where feasible, biodegradable and reusable products should be prioritized over disposable and contaminant, while maintaining infection prevention standards. (see also reuse)
- Creating a Healthy Environment in dialysis areas and ICU, controlling noise pollution and light levels promote patient rest and recovery and reduce energy use.

More specifically in critical care nephrology, the most remarkable way to avoid waste production is Prevention. In medicine, prevention is key for reducing resource use and waste production (15). The highest saving of resources comes from the absence of need to use. In critical care nephrology preventing AKI and reducing progression to severe stages requiring RRT is arguably the most effective sustainability strategy (16, 17).

Beside classical prevention strategies described elsewhere, artificial intelligence (AI) and machine learning (ML) tools have shown promise in predicting AKI development, allowing for timely interventions that preserve kidney function and reduce the need for resource-intensive therapies (18–20).

Predictive AI models, using real-time electronic health record data, can identify patients at risk for AKI with high sensitivity, supporting targeted preventive strategies. By reducing RRT demand, AI contributes indirectly to lowering the ICU’s environmental footprint, while improving patient outcomes (21).

When AKI is already severe and requires RRT, dynamic adjustment of CRRT and IHD doses, based on evolving patient needs (vg adjusting doses, dialysate fluid rate, time of therapy). Personalization of RRT reduces volume of replacement fluid use and associated waste (water, plastic bags, carbon footprint of transportation of fluids, etc) without compromising care quality (8, 11, 22).

#### Reuse

Although not always feasible, some elements can be reused, for example fabric coats, scrubs or hats and to avoid single use plastic non-recyclable clothes. Despite the reduction of plastic non-reusable waste, it implies the use of water for washing and sterilizing when needed.

In CRRT there is the opportunity of minimizing some plastic waste by reusing effluent bags within the same session after proper drainage. At an average CRRT dose of 25 mL/kg/h, the effluent requires 6–7 bags per day. Reusing those bags allows the number of bags to diminish to 1–2 per day.

#### Recycle wisely

In 2012, the United States produced approximately 31.75 million tons of plastic waste, but only 8.8% of that amount was recycled. This level of recycling translates into a reduction of greenhouse gas emissions equivalent to removing about 670,000 cars from the roads each year, or 3.2 million metric tons of CO₂ emissions avoided (23).

Many recyclable plastics used in catheter implantation or in CRRT or HDI settings are not in contact with the patient or his environment. Discarding those clean elements in adequate recycle bins permit it to be recycled in adequate local centers (9, 12) and diminish the amount of incinerable waste from contaminated bins (24). (See table 1) The use of incineration for health care wastes has an enormous ecological impact due to the production of dioxins, furans, and co-planar polychlorobiphenyls (PCBs) in the process. Those subproducts of combustion are toxic and hazardous when not managed properly (25).

**Table 1:** Plastic types in medical waste: identification and recyclability.

Code	Plastic type	Common medical uses	Recyclability
1	PET / PETE (Polyethylene Terephthalate)	IV solution bottles, disposable medication containers	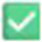 Recyclable in most programs
2	HDPE (High-Density Polyethylene)	Rigid containers for antiseptics, bleach, sharps bins	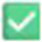 Recyclable and widely accepted
3	PVC (Polyvinyl Chloride)	IV tubing, oxygen masks, blood bags, catheters	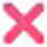 Not recyclable — usually incinerated
4	LDPE (Low-Density Polyethylene)	Surgical drapes, tubing coatings, protective sheeting	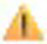 Sometimes recyclable (check locally)
5	PP (Polypropylene)	Syringe barrels, specimen cups, pill bottles, autoclave packaging	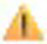 Recyclable in select programs
6	PS (Polystyrene)	Disposable trays, test tube racks, insulated coolers for samples	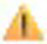 Limited recyclability — varies widely
7	Other / Mixed Plastics	Multi-layer pouches, equipment casings, composite items	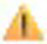 Rarely recyclable — complex materials

In healthcare settings, plastic waste may be classified as regulated medical waste (RMW) depending on contamination. Clean, unused, or packaging materials are more likely to qualify for municipal recycling, especially if properly segregated. [Adapted from: Joseph et al. (24)].

Recyclability depends on kind of material and epidemiological risks associated with the waste. Is crucial to know what kind of plastic fonts can be recycled.

Although hemodialysis filters and tubing are exposed to the patient’s blood and should be classified as hazardous waste requiring incineration, some initiatives—such as that of the non-governmental organization Health Care Foundation in Nepal—have implemented recycling of these components after autoclave disinfection. This strategy is promising; however, it requires adequate resource availability and entails the use of water and electricity, both of which must be carefully assessed (14).

**Figure 1:**
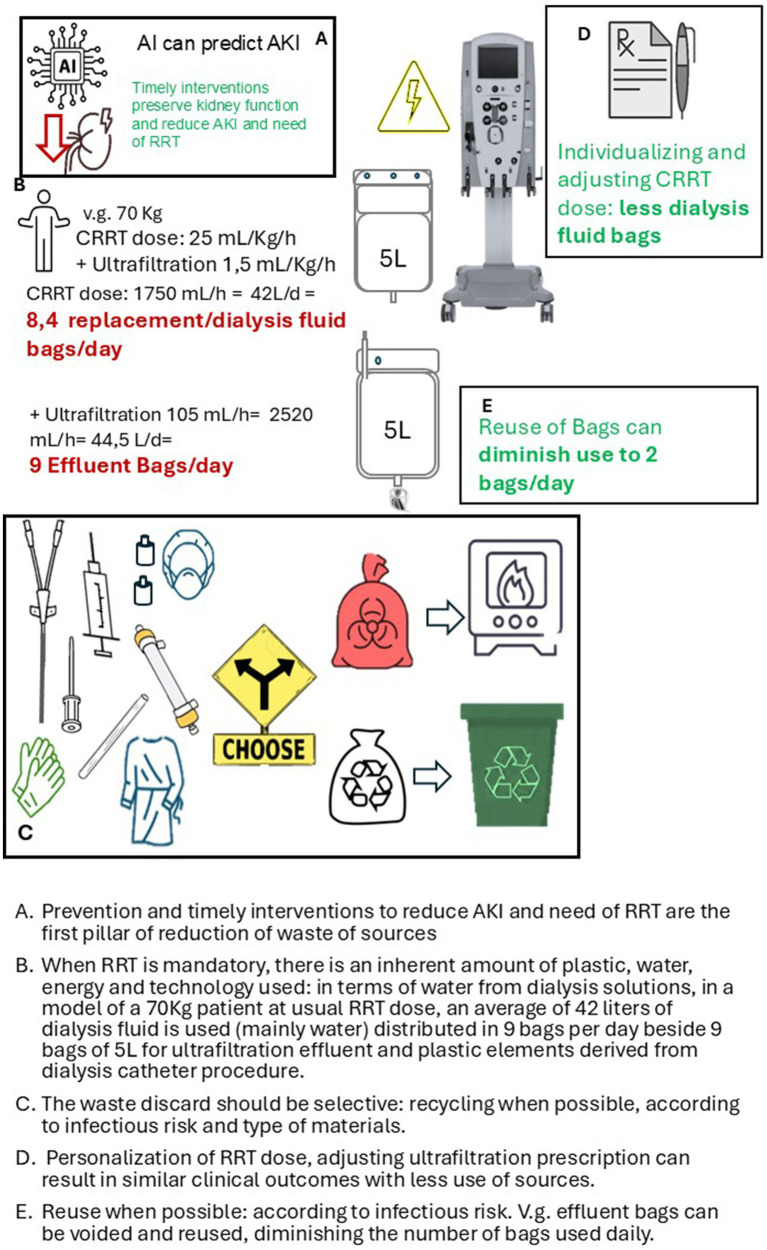
Waste management in critical care nephrology.

To increase the chance of adequate recycling of clean products like paper and plastics, an initiative should be having a recyclable waste collection bin available at the moment of the procedures of setting on CRRT and catheter insertion, but is mandatory to learn about those susceptible to recycle materials (24) (Figure 1).

- Prevention and timely interventions to reduce AKI and need of RRT are the first pillar of reduction of waste of sources
- When RRT is mandatory, there is an inherent amount of plastic, water, energy and technology used: in terms of water from dialysis solutions, in a model of a 70Kg patient at usual RRT dose, an average of 42 liters of dialysis fluid is used (mainly water) distributed in 9 bags per day beside 9 bags of 5 L for ultrafiltration effluent and plastic elements derived from dialysis catheter procedure.
- The waste discard should be selective: recycling when posible according to infectious risk and type of materials.
- Personalization of RRT dose, adjusting ultrafiltration prescription can result in similar clinical outcomes with less use of sources.
- Reuse when posible: according to infectious risk. E.g. effluent bags can be voided and reused, diminishing the amount of bags used daily.

#### Repair

Critical care nephrology uses different kind of dialysis machines. Its appropriate maintenance and repairing are a way to avoid discarding technology that is a source of contamination of soils and water (26, 27).

Repairing and relocating machines can be more cost-effective than purchasing new ones, especially in resource-limited settings (28).

When technology becomes obsolete, the relocation of old functioning machines is advisable, for example donation to developing centers without better available technology.

Adoption of high-efficiency devices and exploration of renewable energy sources for dialysis equipment is a pending area.

#### Rethink

The implementation of Green ICU and critical care nephrology initiatives is crucial for protecting human health, the environment, and reducing economic costs (4–6). Such strategies should involve all personal in the hospital, including administrative areas to coordinate and effectively complement all processes (29, 30).

It is crucial to ensure that healthcare personnel is trained in proper waste management, including associated risks, disposal practices, and minimization, dynamically adjusting processes based on periodic audit with measurable results to assess compliance with waste management norms and procedures (12, 25).

For critical care nephrology, it is time to imagine new strategies, challenge the processes and design alternative sustainable ways to perform changes in daily practice. It is mandatory for the actual generation of nephrologists to redesign the way that medicine is performed, breaking out the resistance to change and the Semmelweis reflex. Historically, prompt acceptation and adaptation to the new circumstances have proven better outcomes.

### Future directions and opportunities

Sustainability in critical care nephrology demands a multidisciplinary approach, combining technological innovation, clinical practice redesign, and cultural change. Key priorities include:

- Generating robust data on the environmental impact of nephrology interventions in ICUs.
- Developing standardized, evidence-based green nephrology protocols adaptable to critical care.
- Scaling AI-driven predictive tools for AKI prevention and RRT demand reduction.
- Integrating sustainability metrics into ICU quality improvement frameworks.
